# The unusual Afrotropical and Oriental leafhopper subfamily Signoretiinae (Hemiptera, Cicadellidae): taxonomic notes, new distributional records, and description of two new *Signoretia* species

**DOI:** 10.3897/zookeys.319.4326

**Published:** 2013-07-30

**Authors:** Daniela M. Takiya, Christopher H. Dietrich, Chandra A. Viraktamath

**Affiliations:** 1 Laboratório de Entomologia, Departamento de Zoologia, Instituto de Biologia, Universidade Federal do Rio de Janeiro, Caixa Postal 68044, Rio de Janeiro, 21941-971, Brazil; 2 Illinois Natural History Survey, University of Illinois, 1816 S. Oak St., Champaign, IL 61820, USA; 3 Department of Entomology, University of Agricultural Sciences, GKVK, Bangalore 560065, India

**Keywords:** Taxonomy, distribution, morphology, new species

## Abstract

The leafhopper subfamily Signoretiinae is redescribed and includes two tribes: Signoretiini Baker and Phlogisini Linnavuori. Redescriptions of included tribes, diagnoses and a taxonomic key to genera are provided. New records for genera of Signoretiinae are as follows: *Phlogis* in Central African Republic, Malaysia and Thailand; *Preta* in Thailand; and *Signoretia* in the Republic of the Congo, Zambia, Thailand, Vietnam, and Taiwan (China). *Signoretia pacifica* is newly recorded from Cameroon. In addition, detailed illustrations of the male genitalia of the previously described species, *Chouious tianzeus*, *Preta gratiosa*,and *Signoretia yangli* are provided; the male genitalia of *Signoretia malaya* are described for the first time; and two new species of *Signoretia* are described, *Signoretia delicata* **sp. n.** from the Philippinesand *Signoretia kintendela* **sp. n.** from the Republic of the Congo.

## Introduction

Signoretiinae is a small, poorly known subfamily of leafhoppers apparently restricted to tropical forests in the Afrotropical and Oriental regions. The group is represented by few specimens in collections and is easily distinguished from nearly all cicadellids by the deeply punctured and enlarged pronotum (see subfamily remarks); other striking morphological features include: face strongly convex with cibarial muscle scars prominent; forewing outer anteapical cell present (vein s present); and hind femur macrosetal formula 2+0+0. Some of these morphological features have led to difficulties in placing the included genera in the Cicadellidae higher classification scheme. Baker (1915) first recognized the group as a subfamily of Stenocotidae (including members of current subfamilies Evacanthinae, Megophthalminae, and Tartessinae) and later (1923) treated Signoretiidae as a family of the Jassoidea. Evans (1947) subsequently placed Signoretiini as a tribe of Aphrodinae. Linnavuori (1978) highlighted the many unusual or unique features present in the group and recognized it as a separate subfamily. For similar reasons, Linnavuori (1979) subsequently recognized an additional subfamily, Phlogisinae, based on a single female specimen of *Phlogis mirabilis* Linnavuori from west Africa.

Dietrich (2005) treated Phlogisinae as a junior synonym of Signoretiinae based on the enlarged, punctate pronotum extended to the scutellar suture in both groups. Subsequent morphology and DNA sequence-based phylogenetic analyses (Dietrich et al. 2010; unpublished data) indicate that these two taxa are sister groups, *i.e*., together form a monophyletic group. Although the relationship of Signoretiinae *sensu lato* to other cicadellid subfamilies was poorly resolved by these analyses, the results indicate that the group belongs to a lineage comprising Cicadellinae, Evacanthinae, and Typhlocybinae.

Signoretiinae *sensu lato* are variable for some morphological characters generally used to define taxa at the subfamily-level in Cicadellidae. For example, the position of the ocelli differs between the two included tribes: in Signoretiini they are found on the crown margin close to the eyes; in Phlogisini they are found on the crown, far from the margin. Similarly, Phlogisinihave hind tibiae with macrosetae in row PD, and forewings with crossveins at the bases of the inner and median anteapical cells; while Signoretiini lack these features. Finally, Phlogisini have a distinct maxillary suture and Signoretiini have a complete longitudinal carina on the frontoclypeus, traits fairly uncommon in leafhoppers. Due to all above-mentioned morphological differences found in these taxa, Phlogisini and Signoretiini are herein treated as valid tribes within Signoretiinae.

**Figure 1.**
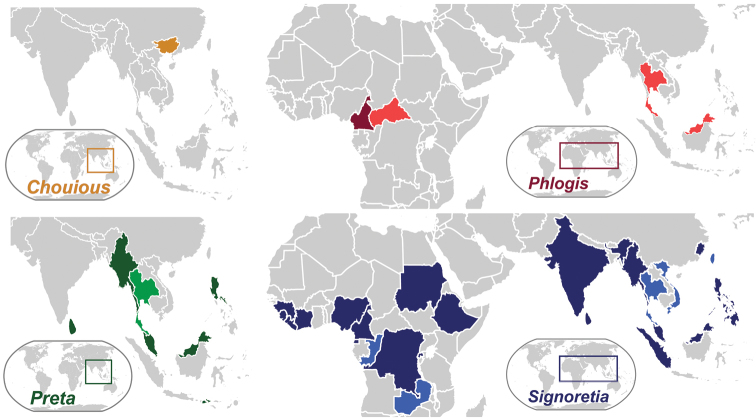
Current distribution of signoretiine genera, *Chouious*, *Phlogis*, *Preta*, and *Signoretia*. Countries marked with a lighter shade are new records given herein.

Signoretiini, as treated herein (Fig. 1), includes *Preta* Distant with two species restricted to the Oriental region and *Signoretia*, occurring both in the Afrotropical and Oriental regions, with 27 species. Phlogisini includes the monotypic genera *Phlogis* Linnavuori from Africa and *Chouious* Yang from China. Although the African Signoretiini were revised by Anufriev (1971) and Linnavuori (1978), the male genitalia have been illustrated and described for only a few species of Oriental Signoretiini. In the present study, we review morphological characters to separate the included tribes and genera of Signoretiinae and a taxonomic key to genera is given. Further taxonomic notes on genera and species of Signoretiinae, new distributional records, descriptions of the male genitalia of *Signoretia malaya* and of two new species of *Signoretia* are also given.

## Materials and methods

Morphological terminology follows Dietrich (2005). Specimens examined are deposited in the following institutions: American Museum of Natural History, New York, USA (AMNH); The Natural History Museum, London, UK (BMNH); Field Museum of Natural History, Chicago, USA (FMNH); Illinois Natural History Survey, Champaign, USA (INHS); Muséum national d’Histoire naturelle, Paris, France (MNHN); Northwest Agriculture and Forestry University, Yangling, China (NWAF); Royal Ontario Museum, Toronto, Canada (ROM); Taiwan Agricultural Research Institute, Taiwan (TARI); and United States National Museum, Washington, DC, USA (USNM). In quotations of type-material labels, a backslash (\) separates lines on a label.

Habitus images were taken with a Digital Lab XLT system by Microptics using a Nikon D1x digital SLR camera and genitalia images were taken with a Q Imaging Micropublisher 3.3 digital camera mounted on an Olympus BX41 compound microscope. Multiple images were combined using the CombineZP software (Hadley 2010). Photographs were modified with Adobe Photoshop and vector illustrations based on the photographs produced with Adobe Illustrator.

## Taxonomy

### Signoretiinae

Baker, 1915

http://species-id.net/wiki/Signoretiinae

Figs 1 –42

Phlogisinae = Linnavuori, 1979

#### Description.

**Figures 2–14.**
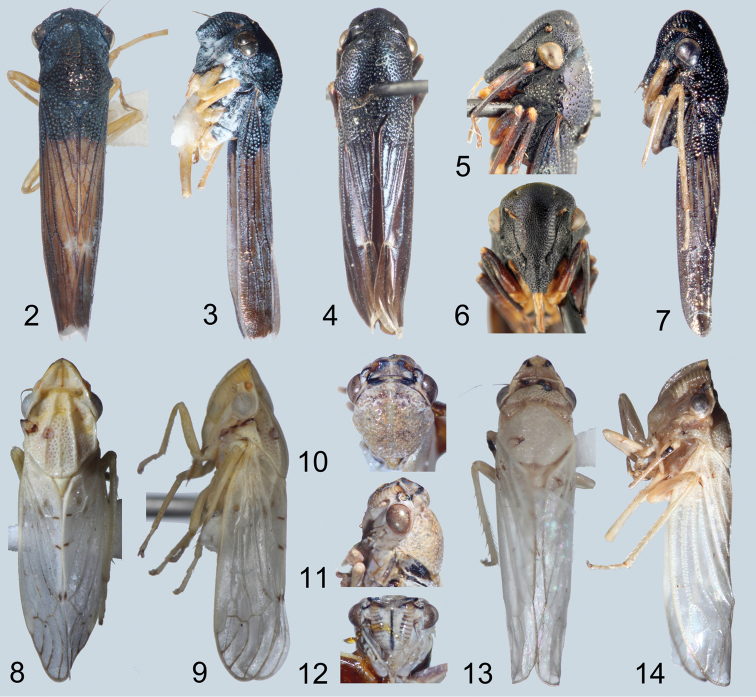
Habitus photographs. **2, 3** *Chouious tianzeus*, dorsal and lateral **4–6** *Phlogis* sp. from Malaysia, dorsal and details of head lateral and face **7** *Phlogis* sp. from Thailand, lateral **8, 9** *Preta gratiosa*, dorsal and lateral **10–12** *Signoretia delicata* sp. n., detail of head, dorsal, lateral, and frontal **13, 14** *Signoretia kintendela* sp. n., dorsal and lateral.

Medium-sized, cylindrical leafhoppers (Figs 2–14). Head (Figs 2–14) broader than pronotum; ocelli visible in dorsal aspect; frontoclypeus expanded with prominent transverse muscle scars; lateral frontal sutures extended ventromesad of ocelli; antennal ledges well developed; antennae subequal to or longer than width of head; anteclypeus convex and tapered from base to apex; lorum short, narrow, well separated from genal margin, partly bordering frontoclypeus; gena slightly emarginate below eyes, exposing proepisternum; rostrum tapered, surpassing front trochanters.

**Figures 15–20.**
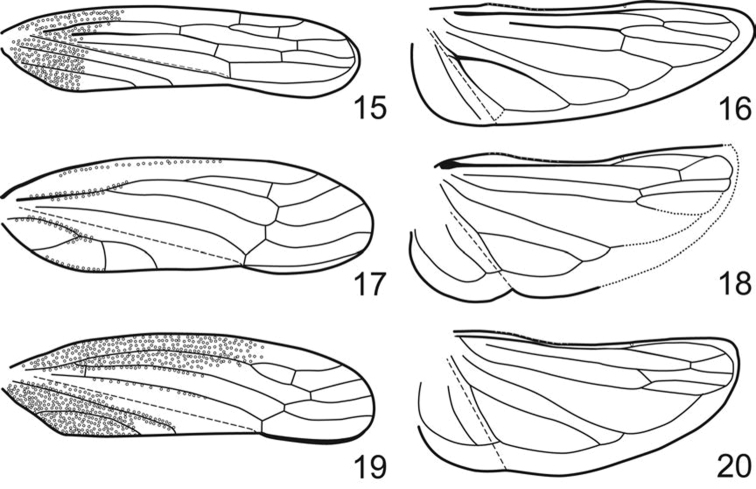
Fore- and hindwing. **15, 16** *Phlogis* sp. from Thailand. **17, 18** *Preta gratiosa*. **19, 20** *Signoretia aureola*.

Pronotum (Figs 2–5, 7–11, 13, 14) greatly enlarged, strongly convex, distinctly punctate, weakly produced anterad, extended posterad to scutellar suture. Forewings (Figs 15, 17, 19) macropterous with venation distinct and opaque sclerotization, if present, limited to basal third; vein R with two (R1 not visible as separate vein) or three branches; crossvein s present (outer anteapical cell closed); inner apical cell elongate, parallel-sided, extended to wing apex. Hind wings (Figs 16, 18, 20) with venation complete; submarginal vein well separated from wing margin. Forelegs with femur with AM1 weakly developed or absent, intercalary row and distal half of AV well differentiated, each with several setae arranged in single row; tibia cylindrical, AD and PD undifferentiated. Hind legs with femur with macrosetal formula 2+0; tibia with macrosetae of dorsal rows reduced in size and number; tarsomere I without dorsoapical pair of macrosetae; pecten with 2 platellae.

Male genital capsule (Figs 21–24, 29–42) with valve articulated or fused laterally to pygofer; pygofer without distinct membranous clefts near base; segment X very large, well sclerotized, with or without processes; subgenital plates digitiform, broadest at base, usually with numerous fine setae dorsally but only rarely with well differentiated macrosetae; connective Y-shaped; style sigmoid; with or without sclerotized dorsal connective or other sclerotized processes between anal tube and aedeagus usually present.

Female ovipositor (Figs 26, 27) elongate, variable in shape and dentition.

**Figures 21–30.**
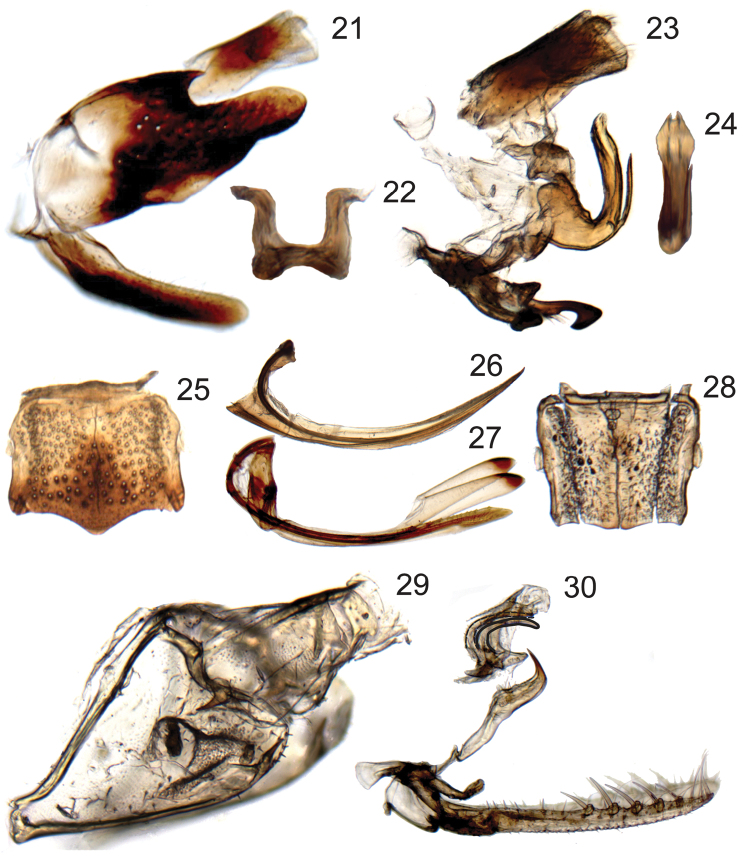
Terminalia of *Chouious*, *Phlogis* and *Preta*. **21–24** *Chouious tianzeus*, male genitalia **21** genital capsule (without anal tube), lateral view **22** dorsal connective, caudal view **23** connective, styles, aedeagus, dorsal connective, and anal tube, lateral view **24** aedeagus caudal view. **25–27** female terminalia of *Phlogis* sp. from Malaysia **25** sternite VII, ventral view **26** first valvula of ovipositor, lateral view **27** second valvifers, second valvulae and gonoplacs of ovipositor, lateral view **28** *Phlogis* sp. from Thailand, sternite VII, ventral view **29, 30** *Preta gratiosa* **29** pygofer and segment X of anal tube, lateral view **30** subgenital plates, connective, styles, and aedeagus, lateral view.

**Figures 31–36.**
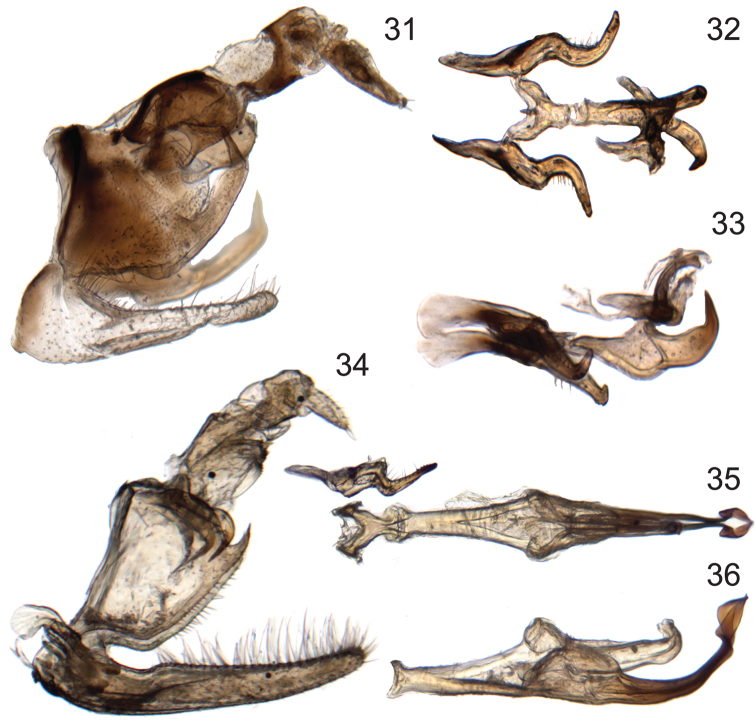
Male genitalia of new species of *Signoretia*. **31–33** *Signoretia delicata* sp. n. **31** genital capsule, lateral view **32** connective, styles, and aedeagus, dorsal view **33** connective, styles, and aedeagus, lateral view **34–36** *Signoretia kintendela* sp. n. **34** genital capsule, lateral view **35** connective, style, and aedeagus, dorsal view **36** aedeagus, ventrolateral view.

**Figure 37–42.**
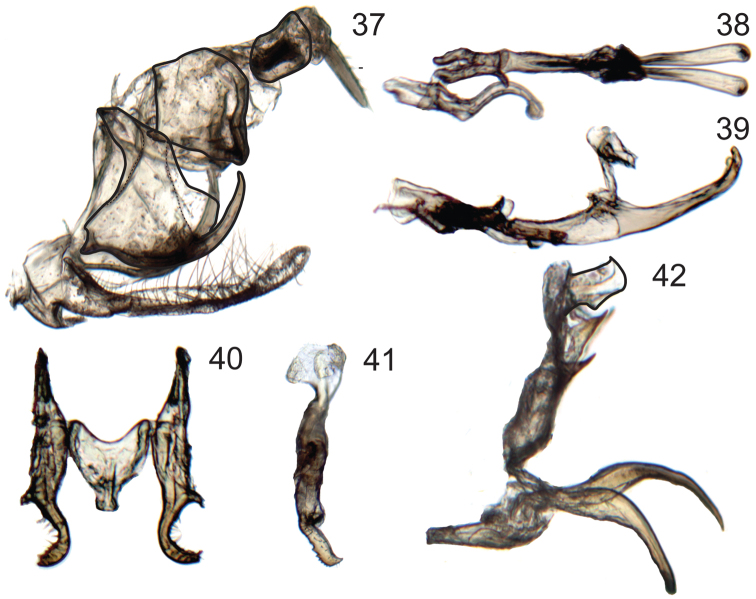
Male genitalia of *Signoretia*. **37–39** *Signoretia malaya*. **37** genital capsule, lateral view **38** connective, styles, and aedeagus, dorsal view **39** connective, styles, and aedeagus, lateral view **40–42** *Signoretia yangli* **40** connective and styles, dorsal view **41** style, lateral view **42** aedeagus, lateral view.

#### Distribution.

Afrotropical and Oriental.

#### Notes.

With the exception of the proconiine sharpshooter genus *Tretogonia* Melichar, 1926 and the recently described dikraneurine (Typhlocybinae) genus *Sweta* Viraktamath & Dietrich 2011, Signoretiinae are the only leafhoppers with fully developed wings that have the pronotum extended to the scutellar suture. Viraktamath and Dietrich (2011) discussed several characters supporting the placement of *Sweta* in Typhlocybinae rather than Signoretiinae. Interestingly all these leafhoppers have the long pronotum distinctly punctate.

Nothing is known about the ecology or feeding behavior of Signoretiinae, although the strongly convex or inflated face suggests that they preferably feed on xylem sap.

#### Key to tribes and genera of Signoretiinae

**Table:** 

1	Dorsal coloration black (Figs 2–7); crown and frontoclypeus without carinae (Figs 2–7); ocelli on crown, each equidistant to adjacent anterior eye angle and other ocellus (Figs 2, 4); forewing with 3 closed anteapical cells (Fig. 15); hind wing submarginal vein not extended onto jugum (Fig. 16); hind tibia with macrosetae in row PD; male pygofer without posteroventral process (Fig. 21); valve fused laterally to pygofer (Fig. 21)	Phlogisini, 2
1’	Dorsal coloration usually white or yellow (Fig. 8–14); crown with transverse basal carina between ocelli (Figs 8, 10, 13); ocelli on crown-face transition, distinctly closer to adjacent eye angle than to median line of crown (Figs 8, 10, 13); frontoclypeus with complete median longitudinal carina (Figs 9, 12, 14); forewing with only outer anteapical cell closed, inner and median anteapical cells open at base (Figs 17, 19); hind wing submarginal vein extended onto jugum (Fig. 18, 20); hind tibia without macrosetae in row PD; male pygofer with posteroventral process (Figs 31, 34, 37); valve not fused laterally to pygofer, articulated by membranous connection (Fig. 31)	Signoretiini, 3
2	Head in profile with lower part of clypeus distinctly produced and angulate, forming shelf over anteclypeus (Fig. 3); frontoclypeus with transverse carina ventrally and median longitudinal carina dorsally (Fig. 3); gena with conspicuous long, pale setae (Fig. 3)	*Chouious*
2’	Head in profile evenly rounded, with lower part of frontoclypeus continuing contour of anteclypeus (Figs 6, 7); face without carinae (Fig. 6); gena without conspicuous long, pale setae (Figs 5, 6)	*Phlogis*
3	Pronotum with complete paired longitudinal carinae (Fig. 8); forewings with claval veins fused for one-third of their distance (Fig. 17)	*Preta*
3’	Pronotum with or without incomplete paired longitudinal carinae at basal third (Figs 10, 13); forewings with claval veins separate (Fig. 19)	*Signoretia*

### Phlogisini

Linnavuori, 1979

http://species-id.net/wiki/Phlogisini

Figs 2–7 , 15, 16 , 21–28

#### Description.

Dorsal coloration dark brown to black (Figs 2–7). Head (Figs 2–7) with crown convex, punctate, carinae indistinct or absent, margins not elevated; ocelli on crown distant from anterior margin, approximately equidistant from eyes and midline; with distinct depression laterad of each ocellus; transition from crown to face rounded; antennal ledges not prominent, evenly rounded; frontoclypeus without median longitudinal carina; anteclypeus with apex emarginated; maxillary suture present.

Pronotum (Figs 2–5, 7) evenly convex, without carinae or deep depressions. Forewings (Fig. 15) with or without R1 and crossveins r-m1 and m-cu2 present. Hind wings with crossvein m-cu perpendicular to CuA; submarginal vein not extended onto jugum. Forefemora with intercalary row strongly arcuate. Hind tibiae with row PD with macrosetae and row PV with some setae blunt-tipped.

Female ovipositor (Figs 26, 27) slender and evenly curved throughout length; first valvulae (Fig. 26) with dorsal sculpturing imbricate along margin and strigate ventrally; second valvulae (Fig. 27) with dorsal teeth small, simple, somewhat irregularly distributed, restricted to distal half. [Female characters of tribe based on *Phlogis*]

Male terminalia (Figs 21–24) with pygofer with apical two-thirds distinctly more sclerotized than base, without posteroventral process; valve fused laterally to pygofer; subgenital plates not extended posteriorly as far as pygofer lobe apex; style without preapical teeth or denticuli; with distinct dorsal connective (separate sclerite connecting aedeagus to anal tube); anal tube segment X with (*Phlogis*) or without (*Chouious*) posterolateral processes.

### Chouious

Yang, 1991

http://species-id.net/wiki/Chouious

Figs 2, 3 , 21–24

Chouious Yang, 1991: 82.

#### Type-species.

*Chouious tianzeus* Yang, 1991.

#### Diagnosis.

Head (Figs 2, 3) coarsely punctate, lateral frontal sutures elevated and carinate; frontoclypeus with median longitudinal carina dorsally, ventral part produced, in contour forming shelf over anteclypeus, with distinct transverse carina, area above carina depressed medially; genae conspicuously pubescent.

#### Notes.

*Chouious* was described based on one new species, *Chouious tianzeus* from south China (Yang 1991). It is similar to *Phlogis* in external morphology, including wing venation and leg chaetotaxy, but differs in the structure of the head, as indicated in the key.

#### Distribution.

China (Guangxi, Yang 1991).

### Chouious tianzeus

Yang, 1991

http://species-id.net/wiki/Chouious_tianzeus

Figs 2, 3 , 21–24

Chouious tianzeus Yang, 1991: 82.

#### Notes.

Forewings of the paratype studied lack crossvein s, but this should be an anomaly of this particular specimen.

#### Distribution.

China (Yang 1991)

#### Material examined.

Male paratype, China, Guanxi, Bose Prov., Tialin Co., 28–30 V 1982, NWAF.

### Phlogis

Linnavuori, 1979

http://species-id.net/wiki/Phlogis

Figs 4–7 , 15, 16 , 25–28

Phlogis Linnavuori, 1979: 684.

#### Type-species.

*Phlogis mirabilis* Linnavuori, 1979.

#### Diagnosis.

Head (Figs 4–7) distinctly but finely punctate; lateral frontal sutures distinct, but not carinate; frontoclypeus evenly convex, without median or transverse carinae, in profile continuing contour of anteclypeus; genae bare.

#### Notes.

*Phlogis* was originally described based on the single female type specimen of *Phlogis mirabilis* from Cameroon (Linnavuori 1979). The genus is readily distinguished from *Chouious* based on the head morphology, as indicated in the key. Three new specimens are herein studied and assigned to this genus, including a male considered to be conspecific to *Phlogis mirabilis* and undescribed females from the Oriental region.

#### Distribution.

Afrotropical: Cameroon (Linnavuori 1979) and Central African Republic [**new record**]. Oriental: Malaysia [**new record**] and Thailand [**new record**].

### Phlogis mirabilis

Linnavuori, 1979

http://species-id.net/wiki/Phlogis mirabilis

Phlogis mirabilis Linnavuori, 1979: 684.

#### Notes.

Previously, the only known specimen of *Phlogis mirabilis* was the female type from Cameroon. A male specimen from Central African Republic is tentatively considered as conspecific to the type-specimen, based on distribution, external morphology, and size (7.4 mm). The male genitalia of this specimen agree with those of *Chouious* in the characters described for Phlogisini. However, this male can be easily distinguished from *Chouious* because its: (1) pygofer lobe lacks the deep concavity on the dorsal margin, (2) aedeagus lacks ventral atrial processes, but has paired apical recurved processes; and (3) anal tube segment X has posterolateral paired processes.

#### Distribution.

Cameroon (Linnavuori 1979) and Central African Republic [**new record**].

#### Material examined.

Central African Republic: male, Boukoko, 15 III 1969, Michel Boulard, MNHN.

### Phlogis spp.

Figs 4–7 , 15, 16 , 25–28

#### Notes.

The only other known specimens of *Phlogis* are two females from the Oriental region, Malaysia and Thailand, examined for this study. Both specimens studied herein (Figs 4–7) agree in most respects with Linnavuori’s original description of the genus, but differ in size (6.4 and 9.5 mm vs. 7.5 mm for *Phlogis mirabilis*) and in some details of body form, indicating that they represent two additional, presumably new species. However, we do not provide formal descriptions, given the meager material available at present. The specimen from Malaysia (Figs 4–6) closely resembles *Phlogis mirabilis*, but is longer (overall length 9.5mm vs. 7.5mm) and differs in the shape of the seventh sternite (Fig. 25). The specimen from Thailand is smaller (6.4mm) and has the shape of the seventh sternite (Fig. 28) very similar to that of *Phlogis mirabilis* (comparison based on photographs of the type-specimen).

#### Material examined.

Malaysia [**new record**]: female, Ranau, 500m, 22–25 I 1959, BMNH. Thailand [**new record**]: female, Petchaburi Kaeng Krachan National Park, km33 helipad, 12°50.177'N,99°20.688'E 735m, Malaise trap, 18–25 V 2009, Sirichai, DNA voucher LH199, INHS.

### Signoretiini

Baker, 1915

http://species-id.net/wiki/Signoretiini

Figs 8–14 , 17–20 , 29 –42

#### Description.

Dorsal coloration pale yellow to white (Figs 8–14; except *Signoretia greeni* Distant, 1908). Head (Figs 8–17) with crown bearing prominent medial, lateral and posterior carinae, margins elevated, punctations indistinct; ocelli closely adjacent to eyes, laterad of submarginal carinae, directly above antennal ledges; transition from crown to face sharp, indicated by transverse carina; antennal ledges prominent, with anterior depression; frontoclypeus with complete median longitudinal carina; anteclypeus with apex truncate; maxillary suture absent.

Pronotum (Figs 8–11, 13–14) with pair of carinae or with median anterior depression. Forewings (Figs 17, 19) without R1 and crossveins r-m1 and m-cu2. Hind wings  (Figs 18, 20) with crossvein m-cu oblique relative to CuA; submarginal vein extended onto jugum. Forefemora with intercalary row weakly arcuate. Hind tibiae with row PD without macrosetae and row PV without blunt-tipped setae.

Female ovipositor sigmoid, broadened near midlength; first valvulae with dorsal sculpturing strigate; second valvulae with dorsal teeth numerous, close-set, and bidentate; toothed area occupying more than half entire length of valvula.

Male terminalia (Figs 29–42) with pygofer with well-developed posteroventral process; valve articulated laterally to pygofer; subgenital plates extended posteriorly beyond pygofer lobe apex; style with preapical teeth or denticuli; dorsal connective absent; anal tube segment X with or without lobes and/or processes at base or more apically; aedeagus divided into ventral paraphyses-like structure articulated to connective consisting of basal preatrium and paired robust processes and dorsal shaft, dorsal and ventral parts may be loosely connected by membrane (all Oriental species) or completely fused to eachother (some African species).

### Preta

Distant, 1908

http://species-id.net/wiki/Preta

Figs 8, 9 , 17, 18 , 29, 30

Preta Distant, 1908: 234.

#### Type-species.

*Preta gratiosa* (Melichar, 1903).

#### Diagnosis.

Head (Figs 8, 9) strongly and angulately produced. Pronotum (Figs 8, 9) with pair of well-developed submedial longitudinal carinae extended entire length. Forewings (Fig. 17) with claval veins fused for short distance near midlength.

#### Notes.

*Preta* is restricted to the Oriental region and currently includes two species, *Preta gratiosa* and *Preta luzonensis* Baker, 1923, the latter known only from the Philippines. It can be easily distinguished from *Signoretia* by its complete paired longitudinal carinae on the pronotum (Fig. 8) and medially fused claval veins of forewings (Fig. 17), as indicated in the key.

#### Distribution.

Indonesia (Sumbawa, Jacobi 1941 *apud* Knight 2010), E. and W. Malaysia (Baker 1923), Myanmar (Distant 1908), Philippines (Luzon, Baker 1923), Singapore (Baker 1923), Sri Lanka (Melichar 1903), and Thailand [**new record**].

### Preta gratiosa

(Melichar, 1903)

http://species-id.net/wiki/Preta_gratiosa

Figs 8, 9 , 17, 18 , 29, 30

Signoretia gratiosa Melichar, 1903: 160.

#### Notes.

Identification based on illustrations of the external morphology and male genitalia of this species (Melichar 1903: plate IV, figs 8a, b; Distant 1908: fig. 148; Baker 1923: plate 1, fig. 8; Anufriev 1971: figs 20–22). Anufriev (1971) did not illustrate the membranous shaft, only the paired ventrally recurved sclerotized processes. Aedeagus shaft imaged here.

#### Distribution.

Indonesia (Sumbawa, Jacobi 1941 *apud* Knight 2010), E. and W. Malaysia (Baker 1923), Myanmar (Distant 1908), Singapore (Baker 1923), Sri Lanka (Melichar 1903); and Thailand [**new record**].

#### Material examined.

Thailand: male, Phetchabun Khao Kho NP, mixed deciduous forest at Ta Phol River, 16°32.561'N, 101°2.479'E, 242m, Malaise trap, 5–12 XI 2006, Somchai Chachumnan & Saink Singhtong, INHS. Malaysia: specimen without abdomen, Kedah Province, Lang Kawi island, 25 V 1975, N. D. Penny, INHS.

### Signoretia

Stål, 1859

http://species-id.net/wiki/Signoretia

Figs 10–14 , 19, 20 , 31 –42

Signoretia Stål, 1859: 289.

#### Type-species.

*Thamnotettix malaya* Stål, 1855.

#### Diagnosis.

Head (Figs 10–14) weakly to strongly produced. Pronotum (Figs 10, 11, 13, 14) with longitudinal carinae absent or, if present, weakly developed and not extended entire length. Forewings (Fig. 19) with claval veins separate throughout length.

#### Notes.

*Signoretia* currently includes 10 Oriental species and 15 Afrotropical species, in addition to the new species described herein. Members of *Signoretia* can be easily distinguished from *Preta* by the lack of paired complete longitudinal carinae on pronotum (Figs 10, 13) and separate claval veins on forewings (Fig. 19). Several nominal species do not have the male genitalia described and illustrated, specially the Oriental ones.

**Distribution.** Afrotropical: Cameroon (Linnavuori 1978), Democratic Republic of Congo (Linnavuori 1978), Equatorial Guinea (Bioko, Anufriev 1971), Ethiopia (Linnavuori 1978), Guinea (Linnavuori 1978), Ivory Coast (Anufriev 1971), Liberia (Linnavuori 1978), Nigeria (Anufriev 1971), Republic of the Congo [**new record**], Rwanda (Linnavuori 1978), Sierra Leone (Anufriev 1971), and Sudan (Linnavuori 1978), and Zambia [**new record**]. Oriental: China (Li 1995), India (Baker 1923), Indonesia (Sumatra, Schimidt 1911 *apud* Knight 2010), E. and W. Malaysia (Baker 1923), Myanmar (Distant 1908), Philippines (Banahao, Luzon, Mindanao, Baker 1915, 1923), Singapore (Baker 1923), Sri Lanka (Distant 1908), Taiwan [**new record**], Thailand [**new record**], and Vietnam [**new record**].

### Signoretia aureola

Distant, 1908

http://species-id.net/wiki/Signoretia_aureola

Figs 19, 20

Signoretia aureola Distant, 1908: 232.

#### Notes.

Identification of specimens at hand is based on Anufriev’s (1971) illustration of the genitalia of a male syntype (BMNH). However, the male from Thailand has an additional larger median black spot near posterior margin of pronotum.

#### Distribution.

Myanmar (Distant 1908) and Thailand [**new record**].

#### Material examined.

Thailand: male, Chiang Mai, Doi Chiang Dao WS Nature trail, 19°24.278'N, 098°55.311'E, 491m, Malaise trap, 7–14 X 2007, Songkran and Apichart, DNA voucher LH193, INHS.

### Signoretia delicata

Takiya & Dietrich sp. n.

urn:lsid:zoobank.org:act:B8DB41FA-7E49-4134-942D-EE3011CC62D6

http://species-id.net/wiki/Signoretia_delicata

Figs 10–12 , 31–33

#### Body length.

Holotype, 6.0 mm

#### Description.

Crown (Figs 10, 11) very short, median length approximately half interocular and three-tenths of transocular width; median longitudinal carina obsolete. Male pygofer (Fig. 31) with caudal margin of lobe weakly sclerotized; ventral appendage robust, spiniform, produced posteriorly beyond pygofer lobe apex, abruptly narrowed and bent dorsad near apex. Valve triangular. Subgenital plates (Fig. 31) extending posteriorly beyond pygofer lobe apex by approximately one-third lobe length, with relatively few long, fine setae dorsally, concentrated near apex. Connective (Fig. 32) Y-shaped; with dorsal median keel and short, slender median anterior lobe. Style (Figs 32, 33) slender, tapering towards apex; apex directed dorsolaterally. Aedeagus (Figs 32, 33) with ventral paraphysis-like structure with pair of robust, tapered, recurved distal processes; dorsal part consisting of pair of parallel dorsolateral arms and median shaft, shaft somewhat depressed and strongly arcuate. Anal tube (Fig. 31) basal section with pair of basal processes short, blunt, extended anteromesad, distal ring weakly sclerotized, retracted into basal section.

**Coloration.** Stramineous to white (Figs 10–12). Crown (Fig. 10) with paired black markings basolaterally, connecting to paired black maculae at apex. Frontoclypeus (Fig. 12) with longitudinal carina black. Legs yellow, coxae and femora infused with fuscous.

#### Etymology.

The species epithet refers to the relatively small size of this species and its delicate habitus.

#### Notes.

This species is described as new because it does not agree with any of the ten previously described Oriental species based on the following combination of characteristics: (1) stramineous dorsal coloration with two pairs of dark markings on crown; (2) median carina on crown absent; (3) each ocellus close to eye for distance of approximately its own diameter; (4) frontoclipeal longitudinal carina not continuing on clypellus; and (5) pronotum longer than wide and without paired incomplete longitudinal carinae on anterior portion, but with very faintly elevated median longitudinal carina. Exceptionally, the above-mentioned characteristics, will not separate *Signoretia delicata* sp. n. from *Signoretia tagalica* Baker, 1915 (another Philippine species described from Luzon and Banahao), with which shares other morphological characters, such as the less produced crown, making the frontoclypeus appear more inflated, and very short outer anteapical cell. Nevertheless, based on the original illustrations and description, *Signoretia tagalica* is larger (male is 6.5 mm) and has a much longer pronotum (more than 4 times the median length of crown) than the species described herein.

The short crown of this new species, shared with other described *Signoretia*, could be viewed as sufficient diagnostic characteristics to place this group in a new genus. Considering that at the moment only a small fraction of Oriental *Signoretia* have the male genitalia described, it would be premature to erect a new genus without reviewing all other described Oriental *Signoretia*.

#### Type material.

Male holotype, “Mindanao: Davao;\ E. slope Mt. Apo,\ Camp Baclayan. Elev.\ 6500 ft. XI-11-1946”, “CMHN-Philippine\ Zool.Exp. (1946–47) \ H.Hoogstraal leg.”, FMNH.

### Signoretia errans

Linnavuori, 1978

http://species-id.net/wiki/Signoretia_errans

Signoretia errans Linnavuori, 1978: 37 (characters in key).

#### Notes.

Linnavuori (1978) included this species in his key and provided illustrations of the male genitalia, but did not include a formal description or list of material examined. However, his figure captions indicate that he examined specimens from the Democratic Republic of Congo (Dingila and Mongbawu). The species is considered valid because Linnavuori (1978) satisfied the criteria of availability by including the species in his identification key for African *Signoretia* and providing illustrations of features that distinguish the species from its congeners.

### Signoretia kintendela

Takiya & Dietrich sp. n.

urn:lsid:zoobank.org:act:ECC53C40-DD5D-483B-988D-4D276DEB9F81

http://species-id.net/wiki/Signoretia_kintendela

Figs 13, 14 , 34–36

#### Body length.

Holotype, 7.4 mm.

#### Description.

Crown (Fig. 13, 14) elongate, median length approximately six-tenths of interocular and half of transocular width; median longitudinal carina well developed. Male pygofer (Fig. 34) with ventral appendage short, spiniform, produced posteriorly beyond pygofer lobe apex. Valve short, rectangular. Subgenital plate (Fig. 34) extended posteriorly beyond pygofer lobe apex by approximately one-third lobe length, with numerous long, fine setae distributed evenly over entire length of dorsum. Connective (Fig. 35) Y-shaped; without dorsal median keel or median anterior lobe. Style (Fig. 28) slender, tapering towards apex; apex directed laterally, with several denticuli preapically. Aedeagus (Figs 35, 36) with ventral paraphysis-like structure with pair of elongate, recurved distal processes expanded preapically; dorsal part without sclerotized apodemes, consisting only of shaft, shaft strongly compressed, extended posterad, abruptly bent dorsad in distal third, distal part sinuate. Anal tube (Fig. 34) segment X with pair of posterolateral lobes each bearing small spine and several denticuli and at base with separately articulated robust process with pygofer, extended ventrad and terminating in anteriorly directed spine.

#### Coloration.

Stramineous to white (Figs 14, 15). Crown (Fig. 14) with paired black markings at base of crown and paired maculae at apex of crown. Etymology. The specific epithet, “kintendela”, means “cicada” in Kongo language (Bentley 1895).

#### Notes.

*Signoretia kintendela* sp. n. shares with other members of the *pacifica* group (Linnavuori 1978) the fusion of the aedeagal shaft to the paraphyses-like structure consisting of the robust and sclerotized paired basal aedeagal appendages and elongate preatrium. If the robust process interpreted herein as arising from the membrane between the anal tube and pygofer lobe was viewed as an anal tube appendage by Linnavuori (1978), then in the *pacifica* group, the new species is more similar to *Signoretia congoensis* and *Signoretia augur* because these species share a single pair of processes at base of the anal tube. Nevertheless, the new species can be distinguished from these and all other *Signoretia* by the shorter ventrocaudal processes of the pygofer lobe and very long and slender rami of basal appendages with sinuous and foliaceous apex.

#### Type material.

Male holotype, “Rep. of Congo: Dept Pool;\ Iboubikro; Lesio-Loun Pk.\ 3°16.196'S, 15°38.267'E\ 330m, malaise trap, A138\ 23.x.2008, Braet & Sharkey”, INHS.

### Signoretia malaya

(Stål, 1855)

http://species-id.net/wiki/Signoretia_malaya

Figs 37–39

Thamnotettix malaya Stål, 1855: 192.

#### Male terminalia.

Pygofer (Fig. 37) with caudal margin of lobe membranous; ventrocaudal process elongate evenly curved and tapered, spiniform, produced posteriorly beyond pygofer lobe apex. Subgenital plates (Fig. 37) extending posteriorly beyond pygofer lobe apex by approximately one-third of lobe length, dorsal surface with numerous long, fine setae evenly distributed throughout length and three macrosetae in longitudinal row near midlength. Connective (Fig. 38) H-shaped, arms subparallel; without dorsal median keel or median anterior lobe. Style (Fig. 38) slender and elongate; apex globose, directed laterally, with few small denticuli. Aedeagus (Figs 38, 39) with ventral paraphyses-like structure with pair of robust, tapered, recurved distal processes; dorsal part consisting of pair of round basolateral lobes and tubular shaft. Anal tube (Fig. 37) with segment X without basal processes and distal margin thickened, terminating ventrally in short lobe.

#### Notes.

Identification based on Stål’s (1859) illustration and collecting locality. Male genitalia previously undescribed.

#### Distribution.

W. Malaysia (Stål 1855), Philippines (Baker 1915), and Singapore (Baker 1923).

#### Material examined.

Malaysia: male, Selangor, Kuala Lumpur, IX 1964, N. L. H. Krauss on *Melastoma malabathricus*, USNM.

### Signoretia pacifica

(Walker, 1858)

http://species-id.net/wiki/Signoretia_pacifica

Tettigonia pacifica Walker, 1858: 357.

#### Notes.

Identification based on Anufriev’s (1971, figs 1–5) and Linnavuori’s (1978, figs 4b, c, d) illustration of the male genitalia. The male holotype is deposited in the BMNH (M. Webb, pers. com.).

#### Distribution.

Cameroon [**new record**]; Democratic Republic of Congo, Guinea, Liberia (Linnavuori 1978); Ivory Coast, Nigeria, and Sierra Leone (Anufriev 1971).

#### Material examined.

Cameroon: male, Litteral, nr. Limbe on road to Bimbia Village, 03°58'192."N, 009°14'16.7"E, 15–30 III 2009, J. R. Cryan & G. J. Svenson, INHS.

### Signoretia yangi

Li, 1995

http://species-id.net/wiki/Signoretia_yangi

Figs 40–42

Signoretia yangi Li, 1995: 6.

#### Notes.

Identification based on illustrations in original description, although male specimens studied differ from original illustration in the shape of the connective. Additionally, distinctive features not mentioned or illustrated in the original publication include the: highly membranous, small and tubular aedeagal shaft; ventral aedeagal processes being separately articulated and densely clothed with microtrichia; and pair of slender ventral spines on segment X extending basad.

#### Distribution.

China: Fujian (Li 1995) and Taiwan [**new record**].

#### Material examined.

Taiwan: male, Nantou Hsien, Tungpu, 1200m, 18–21.x.1982, K. C. Chou & S. C. Lin, TARI; male, Nantou Hsien, Sungkang, 2100m, XI.1985, Malaise trap, K. S. Lin, TARI.

### Signoretia spp.

#### Material examined.

Malaysia: female, Sabah, 1km S. Kundasang, 1530m, 11 IX 1983, G. F. Hevel & W. E. Steiner, USNM. Nigeria: female, Kaduna, Kagoro forest, 29–30 VIII 1973, R. Linnavuori, AMNH [This specimen is similar in external morphology and coloration with the type specimen of *Signoretia astraea* imaged by the AMNH]. Philippines: Mindanao, Davao, Santa Cruz, Badiang, 2000ft, 10 XII 1946, M. Celeston, FMNH. Vietnam [**new genus record**]: female, Thua Thien-Hue: Bach Ma Natl. Pk., edge of stream, ca. 1km along Five Lakes trail 4–16 VI 2000 B. Hubley, Malaise trap, 1200m, subtropical evergreen forest, 16°11'20.1"N, 107°51'08.5"E, DNA voucher PR173, ROM. Zambia [**new genus record**]: female, Northwestern Province, ~15 km N Mwinilunga Lwakera National Forest, 11°34'28.2"S, 24°23'40.1"E, 1445m, 5 XI 2007, Hg-vapor light, J. N. Zahniser, INHS.

## Discussion

As mentioned in the introduction, despite sharing some features unique among Cicadellidae, the two recognized tribes of Signoretiinae show striking differences in major characters of the external morphology. Likewise, the male genitalia also show differences between the two groups. All described Signoretiini have the valve not fused laterally to the pygofer, a strongly developed ventrocaudal process on the pygofer lobe, and long subgenital plates. Males of Phlogisini have, on the other hand, the valve fused laterally to the pygofer, pygofer without processes, and shorter subgenital plates. Furthermore, the aedeagus is articulated with the anal tube by an additional sclerite, the dorsal connective (Hamilton 1983). Signoretiines tend to have one or two pairs of processes arising from the ventral margin of segment X of the anal tube. The presence of these processes is variable within the tribes, and they can originate in different positions and have different shapes, which indicate they may not be homologous structures. For example, all African *Signoretia* and *Preta gratiosa* have processes at the base, while *Signoretia aureola* and *Phlogis mirabilis* bear modifications at a more apical position. However, *Chouious tianzeus* and the remaining Oriental Signoretiini with male genitalia described, *Signoretia delicata* sp. n. and *Signoretia malaya*, have processes strongly reduced or absent.

Linnavuori (1978) in his revision of the African Signoretiini (as Signoretiinae) divided the species of *Signoretia* into two groups: the *pacifica* group including species with the aedeagus shaft “reduced, membranous and more or less concealed by the fused, scoop-shaped appendages” and the *karaseki* group including species with the aedeagus shaft “long and sclerified, distinctly separated from the appendages”. Furthermore, in the key to species, Linnavuori (1978) added that in species of the *karaseki* group the paired basal appendages are long and separate, while in the *pacifica* group they are fused both to each other and, more or less, to the membranous shaft. The definition of these groups based on the sclerotization of the shaft can be confusing, because the degree of apparent sclerotization can vary due to differences in procedures used for preparing the genitalia for study. However, whether the paraphyses-like structure (=basal appendages) is completely fused or not to the base of the shaft seems to be an important taxonomic character, as previously noted by Anufriev (1971). At that the time Anufriev published his study, this character would separate Oriental signoretiines (with an articulated shaft to the basal appendages) from the Afrotropical ones (with a fused shaft and basal appendages). The genitalia of at least some of Linnavuori’s (1978) species from the *karaseki* group do resemble the genitalia of Oriental Signoretiini because of the membranous connection of the highly sclerotized basal appendages to the lightly sclerotized aedeagus shaft. In Oriental species, the shaft can be so short and lightly sclerotized that previous authors have not illustrated it in their genitalia descriptions (Anufriev 1971, Li 1995, see comments above). Although the membranous shaft of members of the Linnavuori’s (1978) African *pacifica* group can be variable, the complete fusion of the paraphyses-like structure to the aedeagal shaft (Fig. 29), seems to be a better character to define this group and has not so far been found in Oriental signoretiines. Additional paired sclerotized cuticular processes were seen associated to this membranous connection in *Signoretia yangi* as triangular tooth-like projections (Fig. 35) and in *Preta gratiosa* (Fig. 23) as curved elongate spiniform projections.

## Supplementary Material

XML Treatment for Signoretiinae

XML Treatment for Phlogisini

XML Treatment for Chouious

XML Treatment for Chouious tianzeus

XML Treatment for Phlogis

XML Treatment for Phlogis mirabilis

XML Treatment for Phlogis

XML Treatment for Signoretiini

XML Treatment for Preta

XML Treatment for Preta gratiosa

XML Treatment for Signoretia

XML Treatment for Signoretia aureola

XML Treatment for Signoretia delicata

XML Treatment for Signoretia errans

XML Treatment for Signoretia kintendela

XML Treatment for Signoretia malaya

XML Treatment for Signoretia pacifica

XML Treatment for Signoretia yangi

XML Treatment for Signoretia
